# Investigating the inequalities in route to diagnosis amongst patients with diffuse large B-cell or follicular lymphoma in England

**DOI:** 10.1038/s41416-021-01523-6

**Published:** 2021-08-13

**Authors:** Matthew J. Smith, Miguel Angel Luque Fernandez, Aurélien Belot, Matteo Quartagno, Audrey Bonaventure, Sara Benitez Majano, Bernard Rachet, Edmund Njeru Njagi

**Affiliations:** 1 Inequalities in Cancer Outcomes Network, Department of Non-Communicable Disease Epidemiology, London School of Hygiene and Tropical Medicine, London, UK; 2 Noncommunicable Disease and Cancer Epidemiology Group, Instituto de Investigación Biosanitaria de Granada, Ibs.GRANADA, Andalusian School of Public Health, Granada, Spain; 3 MRC Clinical Trials Unit, Institute of Clinical Trials and Methodology, University College London, London, UK; 4 CRESS, Université de Paris, INSERM, UMR 1153, Epidemiology of Childhood and Adolescent Cancers Team, Villejuif, France

**Keywords:** Epidemiology, Non-hodgkin lymphoma, Epidemiology, Cancer epidemiology

## Abstract

**Introduction:**

Diagnostic delay is associated with lower chances of cancer survival. Underlying comorbidities are known to affect the timely diagnosis of cancer. Diffuse large B-cell (DLBCL) and follicular lymphomas (FL) are primarily diagnosed amongst older patients, who are more likely to have comorbidities. Characteristics of clinical commissioning groups (CCG) are also known to impact diagnostic delay. We assess the association between comorbidities and diagnostic delay amongst patients with DLBCL or FL in England during 2005–2013.

**Methods:**

Multivariable generalised linear mixed-effect models were used to assess the main association. Empirical Bayes estimates of the random effects were used to explore between-cluster variation. The latent normal joint modelling multiple imputation approach was used to account for partially observed variables.

**Results:**

We included 30,078 and 15,551 patients diagnosed with DLBCL or FL, respectively. Amongst patients from the same CCG, having multimorbidity was strongly associated with the emergency route to diagnosis (DLBCL: odds ratio 1.56, CI 1.40–1.73; FL: odds ratio 1.80, CI 1.45–2.23). Amongst DLBCL patients, the diagnostic delay was possibly correlated with CCGs that had higher population densities.

**Conclusions:**

Underlying comorbidity is associated with diagnostic delay amongst patients with DLBCL or FL. Results suggest a possible correlation between CCGs with higher population densities and diagnostic delay of aggressive lymphomas.

## Introduction

Non-Hodgkin lymphoma is a heterogeneous disease comprising over 60 morphological entities with diverse histological patterns [1]. The most common of which are diffuse large B-cell (DLBCL) and follicular lymphomas (FL), exhibiting an annual rate of 8.2 and 3.3 cases (respectively) per 100,000 people in the UK. These subtypes are relatively common in adults, with incidence increasing amongst older ages [2]. Each of these subtypes has markedly differing treatments and health outcomes [1].

Survival of DLBCL or FL patients in England has steadily increased over the past decades [3, 4], yet the proportion of patients surviving trails that of other European countries [5]. Evidence has highlighted that diagnostic delay (compared to an earlier diagnosis) is associated with a less intensive treatment plan, which then impacts on the chances of survival [6]. Public health policies have aimed to increase awareness, encourage more patient and healthcare system interactions and set targets for earlier cancer diagnosis [7–10].

In the UK, the cancer diagnostic route is defined as the first of eight possible points of contact between the patient and the healthcare system [11]. Emergency diagnosis is defined as a diagnosis of cancer following presentation to an accident and emergency unit, or following an emergency pathway for in/out-patients: it is used as an indicator of diagnostic delay for cancer patients [12]. Underlying comorbidities are known to affect the timely diagnosis of other cancers [13–15]. Comorbidity expressing symptoms similar to cancer may delay the diagnosis: dissimilar symptoms may hasten the cancer diagnosis. For example, some symptoms are present in both lymphomas and other chronic diseases, such as swollen abdomen and fatigue in diabetes [16], chest pain in congestive heart failure [17] and shortness of breath in chronic obstructive pulmonary disease [18]. Furthermore, all three of these diseases are prevalent amongst patients with lymphoma, which could explain misdiagnosis and diagnostic delay [19, 20].

A universal healthcare system (UHS), such as the National Health Service (NHS) in England, aims to provide all residents with access to healthcare [21]. However, variability in health outcomes amongst patients with the same lymphoma still occurs [22, 23]. Clinical Commissioning Groups (CCGs) commission the hospital and community NHS services, and decide on local priorities (informed by general practices), for their respective geographical areas; however, CCGs have shown variability in health outcomes since their inception [24, 25], which may partly explain differences in diagnostic delay.

We aim to assess the association between pre-diagnosed comorbidities and diagnostic delay (i.e., route to diagnosis) amongst patients with DLBCL or FL, accounting for patient sociodemographic characteristics.

## Methods

### Study design, participants, data and setting

We developed a population-based cross-sectional study comprising all patients, aged 18 to 99 years, diagnosed with non-Hodgkin lymphoma (NHL) between January 1, 2005 and December 31, 2013. NHL was coded (C82.0-C85.9) according to the 10th revision of the International Statistical Classification of Diseases and Related Problems (ICD) [26]. Morphology (cell type) and topography (tumour site) were defined using renewed updates of the ICD for Oncology (ICD-O); ICD-O-3 [27] was used for diagnoses up to 2010, and ICD-O-3.1 [28] for diagnoses after 2011. Patients diagnosed with either DLBCL or FL were included in the study and are hereby referred to as subtype (Supplementary Table S1) [26].

Information on patients’ cancer diagnosis was collected by the national cancer registry and analysis service (NCRAS) [29]. The NCRAS contains England national cancer registry data and Hospital Episode Statistics [30] (HES) datasets that are accessed via the Cancer Analysis System [31] (CAS). Cancer registry (CAS dataset) contained information on subtype (morphology), age at diagnosis, ethnicity, gender and date of diagnosis. This was linked to HES, which contained information on patient’s previous hospital admissions, accident and emergency presentations, outpatient appointments.

### Variables

*Route to diagnosis*, obtained from NCRAS, was originally recorded as one of eight routes to diagnosis [11]. Patients with a ‘death certificate only’ route to diagnosis were excluded to remove bias. There is no nationally recognised screening programme for NHL, and no patients were diagnosed via a ‘screen-detected’ route. An ‘unknown’ route to diagnosis was recoded as a missing record. The remaining routes were dichotomised into a binary variable indicating whether the patient was diagnosed following an emergency or elective presentation: elective presentation consisted of patients diagnosed through 2-week-wait, general practitioner referral, inpatient elective and other outpatient.

*Comorbidity status*, based on the Charlson comorbidity index [32] (CCI), was defined as “the existence of disorders, in addition to a primary disease of interest, which are causally unrelated to the primary disease” [33, 34]. Comorbidities were coded within HES according to the International Classification of Diseases, 10th revision (Supplementary Table S2). Previous records of comorbidity were obtained from HES data. Patients with any previous malignancy were removed. For each patient, we defined a time window of 6–24 months prior to cancer diagnosis for a comorbidity to be recorded. A patient’s CCI was determined using an algorithm developed by Maringe et al. [35]. CCI was classified according to the Royal College of Surgeons (RCS) Charlson Score [36], which was categorised into three groups: 0 for no previous comorbidity, 1 for single comorbidity and 2 or more for multimorbidity. We tabulated the prevalence of comorbidity for DLBCL and FL (Supplementary Table S3).

*Stage at diagnosis* is based on the Ann Arbor classification system (CAS dataset) [37]. A lower tumour stage is predictive of a higher survival outcome compared to a higher tumour burden. For NHL subtypes, stages I/II is a criterion for treatment of low tumour stage; stages III/IV is a criterion for treatment of high tumour stage [38]. Therefore, early-stage was dichotomised as I/II, and late-stage as III/IV.

*Deprivation level* (HES dataset) is based on the Lower Super Output Area [39] (*LSOA*) of residence of the patient at the date of cancer diagnosis. An LSOA is a geographical location with a median of 1500 inhabitants. From the Index of Multiple Deprivation [40] (IMD), the income domain was classified into one of five quintiles based on the national distribution of ranked deprivation scores in the 32,844 LSOAs. Each patient was linked with one of the 209 Clinical Commissioning Groups (CCG) where their LSOA resides [41]. Lastly, *ethnicity* (HES dataset) was recorded as either white or other.

### Statistical analysis

We described the study population, tabulated the patient characteristics with diagnostic delay markers (route to diagnosis), and calculated unadjusted odds ratios (and 95% confidence intervals [CI]) with Wald test *P* values.

We conducted analysis for DLBCL and FL separately. Univariable independent logistic regression models were used to explore the crude association between the route to diagnosis and each of the patient characteristics. Then, multivariable generalised linear mixed-effect models (GLMM) were used to account for the dependency between patients *j* = 1,…,*n*_*i*_ from CCG *i* = 1,…,209. The GLMM model for the route to diagnosis was defined as

$${{{\mathrm{logit}}}}\left( {\pi _{ij}} \right) = \beta _0 + b_i + \beta _1A_{ij} + \beta _2G_{ij} + \beta _3E_{ij} + \mathop {\sum }\limits_{k = 2}^5 \beta _{4k} \cdot D_{ijk} + \mathop {\sum }\limits_{k = 2}^3 \beta _{5k} \cdot C_{ijk}$$

where $b_i\sim N(0,\sigma _b^2)$. The patient, and tumour, characteristics were age (*A*), gender (*G*), ethnicity (*E*), deprivation (*D*) and comorbidity score (*C*).

The model was estimated using maximum likelihood. Likelihood-ratio tests were used to compare between models with and without each covariate and for linear trend. Note that these and subsequent estimates are for any given CCG as results from logistic mixed-effects models have cluster-specific interpretation [42–45]. Empirical Bayes estimates of the random effect $\widehat {b_i}$ were used to explore the between-CCG variability in the odds of the emergency route to diagnosis. The random-effect variance parameter was tested for using a mixture of Chi-squares with 0 and 1 degrees of freedom [42, 43]. The mixture of the Chi-square test is a likelihood-ratio-type test, where an appropriate reference distribution is used to account for the fact that the null hypothesis in this case is at the boundary of the parameter space [42, 46]. Combining likelihood-ratio tests after multiple imputation requires derivation of a particularly modified likelihood-ratio test statistic, which is compared with a particularly derived reference distribution. For tests of fixed-effect parameters, the relevant methodology exists [47]. We are not aware of the existing corresponding methodology for combining after multiple imputation likelihood-ratio-type tests for random-effect variance parameters.

### Missing data analysis

Variables with missing data were the outcome (route to diagnosis [DLBCL: 1.9%, FL: 2.1%]), and ethnicity [DLBCL: 22.8%, FL: 24.9%]. Using logistic regression models, we explored the missing data mechanism for each partially observed variable. The imputation model included all fully- and partially observed covariates and the cluster variable indicator. To reduce potential bias [47], the auxiliary variables (patient’s vital status, Nelson–Aalen estimate of the cumulative mortality hazard, and stage at diagnosis) were included as, per the missing data indicator model, they were predictive of the chance of missing values and, as per subject matter knowledge, associated with the underlying values themselves [48]. We used the latent normal joint modelling multiple imputation approach, under a missing at random assumption, and generated ten imputed datasets. The multilevel logistic regression models for each outcome were fitted to each of these datasets and results combined using Rubin’s rules [49, 50].

We used *R* software for all analysis; the *glmer* function of the *lme4* package was used for generalised linear mixed-effects models, and the jomo [51] package for multiple imputation, which allows imputation of clustered data.

## Results

### Summary statistics

In this study, we included 45,629 patients diagnosed with DLBCL (30,078; 65.9%) or FL (15,551; 34.1%) between January 1, 2005 and December 31, 2013 (Table 1A, B). The prevalence of emergency diagnostic routes amongst those diagnosed with DLBCL or FL was 9683 (34.1%) and 1879 (12.3%), respectively, there was no evidence of a yearly trend. Amongst these patients, the average age at diagnosis was 68.2 and 66.3 years, respectively.

**Table 1:** (A) Summary statistics of the emergency route to diagnosis amongst patients diagnosed with diffuse large B-cell lymphoma (*n* = 30,078) in England during 2005–2013; (B) summary statistics of the emergency route to diagnosis amongst patients diagnosed with follicular lymphoma (*n* = 15,551) in England during 2005–2013.

(A)	Route to diagnosis^a^		cOR^b^	95% CI	*P* value
	Elective *N* = 19,833 (65.9%)	Emergency *N* = 9683 (34.1%)			
Age (mean, s.d.)	67.2 (14.8)	68.2 (15.5)	1.04^c^	1.03–1.06	<0.001
Gender					
Male	10,658 (53.7)	5292 (54.7)	Ref	Ref	Ref
Female	9175 (46.3)	4391 (45.4)	0.96	0.92–1.01	0.139
Ethnicity					
White	14,583 (94.8)	6,898 (92.6)	Ref	Ref	Ref
Minorities	802 (5.2)	549 (7.4)	1.44	1.29–1.62	<0.001
Missing^d^	4448 (22.4)	2236 (23.1)	–	–	–
Deprivation					
Least deprived	4410 (22.2)	1823 (18.8)	Ref	Ref	Ref
2	4455 (22.5)	2105 (21.7)	1.14	1.06–1.23	<0.001
3	4145 (20.9)	2031 (21.0)	1.19	1.10–1.28	<0.001
4	3806 (19.2)	1993 (20.6)	1.27	1.17–1.37	<0.001
Most deprived	3017 (15.2)	1731 (17.9)	1.39	1.28–1.50	<0.001
Comorbidity					
None	17,957 (90.5)	8396 (86.7)	Ref	Ref	Ref
One	970 (4.9)	590 (6.1)	1.30	1.17–1.45	<0.001
Multimorbidity	906 (4.6)	697 (7.2)	1.65	1.49–1.82	<0.001

(B)	Route to diagnosis^e^		cOR^b^	95% CI	*P* value
	Elective *N* = 13,353 (87.7%)	Emergency *N* = 1879 (12.3%)			
Age (mean, s.d.)	63.5 (13.5)	66.3 (14.2)	1.17^c^	1.13–1.21	<0.001
Gender					
Male	6209 (46.5)	962 (51.2)	Ref	Ref	Ref
Female	7144 (53.5)	917 (48.8)	0.83	0.75–0.91	<0.001
Ethnicity					
White	9459 (94.9)	1399 (94.8)	Ref	Ref	Ref
Minorities	510 (5.1)	77 (5.2)	1.02	0.80–1.31	0.870
Missing^d^	3384 (25.3)	403 (21.5)	–	–	–
Deprivation					
Least deprived	3100 (23.2)	375 (20.0)	Ref	Ref	Ref
2	3040 (22.8)	405 (21.6)	1.10	0.95–1.28	0.205
3	2857 (21.4)	375 (20.0)	1.09	0.93–1.26	0.292
4	2462 (18.4)	412 (21.9)	1.38	1.19–1.61	<0.001
Most deprived	1894 (14.2)	312 (16.6)	1.36	1.16–1.60	<0.001
Comorbidity					
None	12,410 (92.9)	1667 (88.7)	Ref	Ref	Ref
One	536 (4.0)	95 (5.1)	1.32	1.05–1.65	0.015
Multimorbidity	407 (3.1)	117 (6.2)	2.14	1.73–2.65	<0.001

*cOR* crude odds ratio, *CI* confidence interval.

^a^In all, 562 (1.9%) missing route to diagnosis records.

^b^Crude odds ratios for emergency vs elective.

^c^Increase in odds of the emergency route for each 10-year increase in age.

^d^ Proportions of missing records amongst all ethnicity records (including observed records).

^e^In all, 319 (2.1%) missing route to diagnosis records.

Percentages may not sum to 100% due to rounding.

The prevalence of emergency diagnostic routes (compared to elective) was higher amongst FL males, ethnic minorities in DLBCL, and those living in the most deprived areas (both DLBCL and FL). The emergency route, compared to elective, was more common amongst those with multimorbidity: DLBCL (7.2% vs 4.6%, respectively) and FL (6.2% vs 3.1%, respectively). Similarly, for both DLBCL and FL, an increase in the crude odds of the emergency route to diagnosis was strongly associated with an increase in age and living in most deprived areas, while for an ethnic minority it was observed in DLBCL only. There was an increase in the odds of the emergency route to diagnosis with each increase in deprivation level.

### Multivariable mixed-effect logistic regression models

Table 2A, B shows the results from the multivariable GLMM for odds of the emergency route to the diagnosis of DLBCL and FL, respectively. For both DLBCL and FL, under complete case analysis, we found that for any given CCG, the presence of comorbidity was associated with the emergency route to diagnosis: the association was largest amongst those with a comorbidity status of two or more (Table 2A, B). Living in more deprived areas was strongly associated with the emergency route to diagnosis.

**Table 2:** (A) Multivariable GLMM for the odds of the emergency route to diagnosis in (a) complete case analysis, (b) multiple imputation amongst patients (*n* = 30,078) diagnosed with diffuse large B-cell lymphoma in England during 2005–2013; (B) multivariable GLMM for the odds of the emergency route to diagnosis in (a) complete case analysis, (b) multiple imputation amongst patients (*n* = 15,551) diagnosed with follicular lymphoma in England during 2005–2013.

(A)	(a) Complete case analysis (*n* = 22,832)			(b) After multiple imputation (*n* = 30,078)		
	OR	95% CI	*P* value	OR	95% CI	*P* value
Age^a^	1.03	1.02–1.04	0.002	1.05	1.04–1.06	<0.001
Gender						
Male	Ref	Ref		Ref	Ref	
Female	0.95	0.90–1.01	0.082	0.95	0.91–1.00	0.061
Ethnicity						
White	Ref	Ref		Ref	Ref	
Minority	1.44	1.28–1.62	<0.001	1.42	1.26–1.60	<0.001
Deprivation						
Least deprived	Ref	Ref		Ref	Ref	
2	1.14	1.04–1.24	0.003	1.13	1.05–1.22	0.001
3	1.18	1.08–1.29	<0.001	1.17	1.08–1.27	<0.001
4	1.23	1.12–1.34	<0.001	1.23	1.14–1.34	<0.001
Most deprived	1.24	1.13–1.36	<0.001	1.32	1.21–1.43	<0.001
Comorbidity						
None	Ref	Ref		Ref	Ref	
One	1.26	1.12–1.41	<0.001	1.27	1.14–1.41	<0.001
Multimorbidity	1.58	1.41–1.78	<0.001	1.56	1.40–1.73	<0.001
Variance of RE (s.d.)	0.007 (0.09)	–	–	0.008 (0.09)	–	–

(B)	(a) Complete case analysis (*n* = 11,445)			(b) After multiple imputation (*n* = 15,551)		
	OR	95% CI	*P* value	OR	95% CI	*P* value
Age^a^	1.15	1.12–1.17	<0.001	1.17	1.15–1.19	<0.001
Gender						
Male	Ref	Ref		Ref	Ref	
Female	0.76	0.68–0.85	<0.001	0.80	0.73–0.89	<0.001
Ethnicity						
White	Ref	Ref		Ref	Ref	
Minority	1.03	0.80–1.32	0.835	1.03	0.81–1.29	0.833
Deprivation						
Least deprived	Ref	Ref		Ref	Ref	
2	1.16	0.98–1.38	0.084	1.11	0.95–1.29	0.190
3	1.09	0.92–1.30	0.312	1.07	0.92–1.24	0.396
4	1.42	1.20–1.69	<0.001	1.38	1.18–1.61	<0.001
Most deprived	1.38	1.14–1.66	<0.001	1.39	1.18–1.64	<0.001
Comorbidity						
None	Ref	Ref		Ref	Ref	
One	1.18	0.92–1.51	0.190	1.19	0.94–1.49	0.143
Multimorbidity	1.78	1.40–2.26	<0.001	1.80	1.45–2.23	<0.001
Variance of RE (s.d.)	0.016 (0.128)	–	–	0.017 (0.130)	–	–

*OR* odds ratio, *CI* confidence interval.

^a^Increase in odds of emergency route to diagnosis for each 10-year increase in age at diagnosis.

After multiple imputation (Table 2A, B), there were similar conclusions to the complete case analysis. Amongst patients from the same CCG, having a comorbidity score of 2 or more, compared to no comorbidity, was strongly associated with an emergency route to diagnosis (DLBCL: OR 1.56, CI 1.40–1.73; FL: OR 1.80, CI 1.45–2.23). There was weak evidence of a trend for deprivation and comorbidity index amongst DLBCL (*P* = 0.054 and *P* = 0.060, respectively); however, there was no evidence of a trend amongst FL (*P* = 0.206 and *P* = 0.113, respectively).

Using a mixture of Chi-square tests with 0 and 1 degree of freedom (i.e. half the *P* value from a Chi-square with 1 degree of freedom), we found strong evidence of between-CCG variability in the odds of the emergency route to diagnosis (DLBCL: *P* < 0.005; FL: *P* < 0.001). The variance of the CCG random effects of the models for DLBCL and FL indicated some heterogeneity between CCGs in routes to diagnosis.

**Fig. 1:**
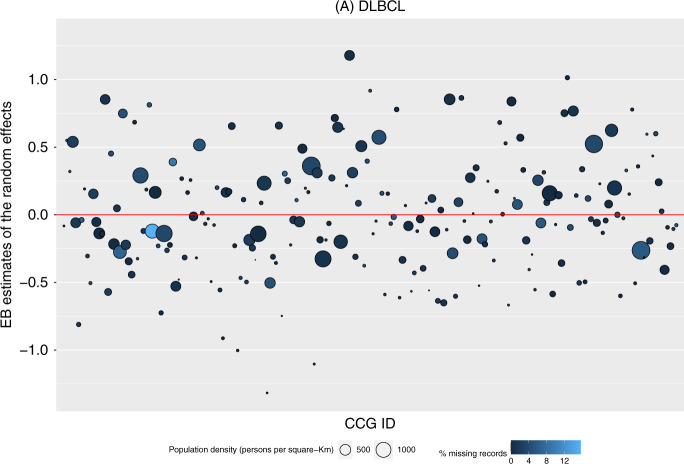
Variance of diagnostic delay of diffuse large B-cell lymphoma amongst clinical commissioning groups. Empirical Bayes estimates of the random effects from the model for the route to diagnosis, by each Clinical Commissioning Group, amongst patients (*n* = 30,078) diagnosed with diffuse large B-cell lymphoma in England during 2005–2013.

**Fig. 2:**
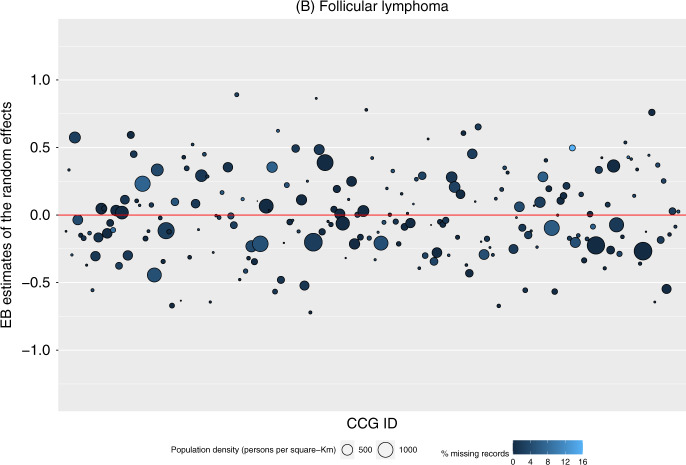
Variance of diagnostic delay of follicular lymphomas amongst clinincal commissioning groups. Empirical Bayes estimates of the random effects from the model for the route to diagnosis, by each Clinical Commissioning Group amongst patients (*n* = 15,551) diagnosed with follicular lymphoma in England during 2005–2013.

We graphically illustrate, from our analysis accounting for both clustering and missing data, the Empirical Bayes (EB) estimates of the CCG random effects for odds of the emergency route to diagnosis (Figs. 1 and 2). These are used to explore the between-CCG variability. A positive EB estimate indicated a higher probability of emergency route to diagnosis for a patient from that CCG in comparison to a patient who has similar observed characteristics but from a CCG with either a less positive, or a negative EB estimate. For DLBCL, there are possibly a few outlying CCGs with the lowest probabilities, and possibly an outlying one with the highest probability. For FL, there are possibly a few outlying ones with the highest probabilities. To explore possible patterns, the size of the markers were weighted by the population density for the respective CCG and have a lighter shade for a higher proportion of missing records of the route to diagnosis.

For DLBCL (Fig. 1), the results show a slight pattern such that there were more CCGs with a larger population density (larger-sized markers) that had a higher probability for their patients being diagnosed through an emergency route to diagnosis (markers with EB estimates above 0). There was no apparent pattern for patients with FL (Fig. 2).

## Discussion

We aimed to assess the association between comorbidity status and a marker of diagnostic delay (route to diagnosis), amongst patients diagnosed with non-Hodgkin lymphoma, adjusting for patient and healthcare pathway characteristics.

We found that comorbidity status was significantly associated with the emergency route to diagnosis, after adjusting for age, gender, ethnicity and deprivation and accounting for clustering due to CCG did not explain the relative difference. The more severe the comorbidity score, and those living in more deprived areas, increased the odds of the emergency route to diagnosis. Our results are consistent with previous findings of an increase in the probability of emergency route to diagnosis [6, 52], and, in other countries and for other cancers, comorbidities were associated with diagnostic delay [53]. Similar results were found amongst studies investigating colon cancer [54, 55]. Since the proportion of patients with emergency route remains stable over calendar time, this phenomenon is not thought to be time-dependent.

Deprivation level was a strong independent predictor of the route to diagnosis after adjusting for comorbidity and other factors (Table 2A, B); however, accounting for clustering increased the strength of the association for patients living in more deprived areas. This suggests that the difference in diagnostic delays between deprivation groups is partly explained by unobserved, and possibly unmeasured, characteristics of CCGs. A characteristic of CCGs, not explored in this study but for other cancers, could be accessed to the healthcare system (e.g., accessibility to a GP appointment) [56]. Previous studies [57] have found delays in diagnosis since first symptoms and suggested introducing rapid access to lymph node diagnostic clinics [58] and providing: less variability in the number of GP appointments attended before a diagnosis [59, 60], clearer definitions of symptoms [61], and appropriate patient-oriented information when previous investigations rule out cancer [15]. These unmeasured characteristics of CCGs could explain the large between-CCG variation in outcomes. In the United States, and for other malignancies, physician supply is associated with early detection of breast cancer [62], and higher primary care physician density is associated with a lower incidence of late-stage colorectal cancer [63].

Contrary to the assurances of a universal healthcare system, such as the NHS, our results suggest inequitable access to healthcare services between CCGs (i.e., more densely populated CCGs appear to have patients with a greater chance of diagnostic delay compared to less densely populated CCGs). Patients diagnosed through the emergency route are patients that either could not access a GP appointment or the GP appointment was inconclusive: during this waiting time, cancer can progress and the patient admitted themselves to the emergency department. Inequalities may be due to a combination of competing demands and a lack of clinical guidance regarding symptoms. However, lack of clinical guidance would be a non-differential misclassification and this would not explain the inequalities in the emergency route amongst patient characteristics.

Our results challenge previous research that did not find evidence of a difference in diagnostic delay between deprivation levels using unadjusted analyses; although, previous studies were based on a smaller sample size that were potentially underpowered in comparison to our study [6]. We highlight that deprivation is predictive of the diagnostic route if analyses do not account for CCGs that widely differ, among other dimensions, in healthcare provision [64]. Furthermore, the late lymphoma stage at diagnosis seems associated with poorer survival. Evidence is limited due to the extended use of the FL and DLBCL International Prognostic Indices (FLIPI and IPI, respectively) and for lymphoma prognosis and survival outcomes. The indices, in addition to the lymphoma stage, integrate other prognostic factors such as serum lactate dehydrogenase, the number of nodal site involvement, patient ages, and haemoglobin. Evidence shows that a higher index score, and thus a higher stage, is associated with poorer health outcomes and survival: highlighting the necessity of prompt management among patients at an advanced stage [65].

We graphically illustrated that patients living in CCGs with more dense populations have a higher probability of emergency route to diagnosis. To our knowledge, there is yet no research into the relationship between population density and diagnostic delay of cancer in England. This study shows that NHL patients living in CCGs with higher population densities have a higher probability of emergency route to diagnosis. On one hand, deprivation tends to be correlated with high population density in England [66], and is also associated with higher use of emergency services [67]. On the other hand, population density is independently associated with high emergency calls [68]. This could be because highly dense areas accumulate high demands that are not completely covered by available healthcare resources; accordingly, this demand could be exacerbated by the association between deprivation and the prevalence of comorbidities. This association has not been well explored, but it is likely that cancers other than NHL are affected by the association between the prevalence of emergency route to diagnosis and population density. Further research should be conducted to determine the need for greater availability of healthcare services in more populated areas.

Furthermore, there will be differences in the availability and specialisation of cancer-specific resources between CCGs. For example, a CCG may have a specialised centre for breast cancer but not for another cancer. Additional analyses are needed to provide a full interpretation of these results. Densely populated areas may be associated with populations from less favourable backgrounds and potentially higher pressure on the healthcare system. CCGs were established from the Health and Social Care Act 2012 and replaced Primary Care Trusts (PCTs). However, CCGs and PCTs were constructed based on administrative boundaries, and the population size of CCGs are similar to the PCTs they replaced. Since 2013, the number of CCGs have reduced due to mergers [69], and the proportion of late-staged lymphomas has increased [70], possibly indicating competition for healthcare services.

Our study is strengthened by the large population-based sample capturing all patients with a diagnosis of DLBCL and FL between 2005 and 2013. To date, this is the largest study of diagnostic delay amongst patients with NHL. Patients were diagnosed according to the latest (ICD-O-3) well-defined WHO cancer classifications, and through a linkage of databases we obtained reliable information on comorbidity diagnosis prior to, and likely independent of, cancer. The objective data sources provide information on patients that is gathered prospectively, preventing differential misclassification.

Despite the lack of well-defined guidance on which comorbidity index is the gold standard depending on the setting of the study, Charlson comorbidity index (CCI) is one of the most commonly used comorbidity indices in population-based cancer epidemiology [71]. We used the Royal College of Surgeons’ adaptation of the CCI, which provides a cancer-specific comorbidity indicator, and is advantageous in comparison to other indices that measure underlying comorbidities as independent from each other [32, 71, 72]. Computed algorithms were used to define comorbidity status, which strengthens the reliability of this study [35].

In this study, we had missing data in two dimensions: route to diagnosis (the outcome) and explanatory variables. Missing data in outcomes present less complexity when using a likelihood-based analysis such as a generalised linear mixed model, as the ignorability property assures the validity of results from analysis of the complete cases, under a missing at random mechanism [42, 47]. With missing data additionally in explanatory variables, analyses are more complex, as multiple imputation is in general needed to achieve validity of results under a missing at random mechanism, if the outcome is included in the missingness mechanism for these variables. Research in missing data has shown that multiple imputation has the potential to mitigate bias and loss of efficiency; whether multiple imputation provides gains over a complete case analysis cannot be simply determined from the proportion of incomplete cases in a single variable. Indeed, potential benefits from multiple imputation depend on factors such as whether missing data occur in the explanatory variable of interest or covariates, and interrelationships between the variables [73]. Lee and Carlin [73] and White and Carlin [74] have highlighted the importance of conducting both a complete case analysis and an analysis after multiple imputation, and carefully compare results. We used the latent normal joint modelling multiple imputation approach under a missing at random assumption to account for the missing ethnicity and route to diagnosis. This approach allows imputation of a mix of variable types, while accounting for multilevel structures arising from clustering of patients [47, 75, 76]. As with all missing data problems, it is impossible to distinguish between a missing at random and a missing not at random mechanism based on the observed data [47, 77–79]. Follow-up work will therefore involve assessing the sensitivity of our results to departures from the missing at random mechanism, by imputing under a missing, not at random assumption.

A limitation of this study is that route to diagnosis does not entirely encapsulate the patient’s multifaceted experiences along the healthcare pathway prior to a cancer diagnosis. Information on performance status and education were not available but may have contributed to differences in diagnostic delay. Firstly, distinct from having comorbidity, performance status measures the patient’s ability to carry out everyday tasks, such as reaching the healthcare system, which may contribute to diagnostic delay [6]. Secondly, the low average time allocated for each GP appointment requires the patient to use the English language efficiently and describe important symptoms in a concise manner, which may hasten the cancer diagnosis [80].

## Conclusion

Patients with DLBCL or FL are more likely to experience an emergency route to diagnosis if they have underlying comorbidity. Differences in diagnostic delay indicators between deprivation levels are minimally explained by comorbidity status, and are further explained by differences in the healthcare provisions between clinical commissioning groups (CCG). DLBCL patients living in CCGs with higher population densities have a higher probability of emergency route to diagnosis.

## Supplementary information

Supplementary material

Reproducibility checklist

## Data Availability

The data that support the findings of this study are available via application to the Public Health England Office for Data Release, but restrictions apply to the availability of these data.
