# Supplementary material for: Investigating the inequalities in route to diagnosis amongst patients with diffuse large B-cell or follicular lymphoma in England
Source: Br J Cancer. 2021 Aug 13;125(9):1299–307. doi: 10.1038/s41416-021-01523-6 (PMC8548410; doi:10.1038/s41416-021-01523-6)
Supplement: Supplementary file 1 — Supplementary material [file 41416_2021_1523_MOESM1_ESM.docx]

# **Appendix**

This page is intentionally blank. Please move to the next page.

**Supplementary Table S1:** Distribution of non-Hodgkin lymphoma subtypes for patients diagnosed from 2005-2013, with respective morphology and topography ICD-O-3 codes. DLBCL (index 4) and Follicular (index 6) lymphomas were included in this study.

|  | | | | | | |
| --- | --- | --- | --- | --- | --- | --- |
| **Index** | **Site group (subtype)** | **Grade** | **Topography** | **Morphology** | **n** | **%** |
|  |  |  |  |  |  |  |
| 1 | CLL/SLL* | Indolent | C82.0-C85.9 | 9670, 9823 | 4,043 | 4.78 |
| 2 | Waldenstrom macroglobulinemia | Indolent | C82.0-C85.9 | 9761 | 2,453 | 2.90 |
| 3 | Mantle cell | Indolent | C82.0-C85.9 | 9673 | 3,549 | 4.20 |
| 4 | Diffuse large B-cell | Aggressive | C82.0-C85.9 | 9680, 9688, 9737-9738 | 30,750 | 36.39 |
| 5 | Burkitt | Aggressive | C82.0-C85.9 | 9687, 9826 | 1,077 | 1.27 |
| 6 | Follicular | Indolent | C82.0-C85.9 | 9690-9691, 9695, 9698 | 15,624 | 18.49 |
| 7 | Mature T-cell | Aggressive | C82.0-C85.9 | 9702 | 6,066 | 7.18 |
| 8 | Marginal zone B-cell | Indolent | C82.0-C85.9 | 9689, 9699, 9760, 9764, 9699 | 4,615 | 5.46 |
| 9 | Not Otherwise Specified | n/a | C82.0-C85.9 | 9591, 9675, 9735 | 10,308 | 12.20 |
| 10 | Other*** | n/a | C82.0-C85.9 | 9591, 9675, 9735 | 6,019 | 7.12 |
|  |  |  |  |  |  |  |
| Total |  |  |  |  | 84,504 | 100.00** |
| n/a – not applicable; there was no subtype information  * Chronic lymphocytic leukaemia/Small-cell lymphocytic lymphoma  ** Percentages may not equate to 100.0% due to rounding  *** The morphology code specifies these patients are diagnosed with NHL. However, the description states ‘other’; these patients are classified similarly to ‘Not Otherwise Specified’. | | | | | | |

**Supplementary Table S2:** Comorbidities and their diagnostic ICD-10 codes

| **Comorbidity** |  | **ICD-10** |
| --- | --- | --- |
| Myocardial infarction |  | I21.x, I22.x, I25.2 |
| Congestive heart failure |  | I11.0, I13.0, I13.2, I25.5, I42.0, I42.5–I42.9, I43.x, I50.x, P29.0 |
| Peripheral vascular disease |  | I70.x, I71.x, I73.1, I73.8, I73.9, I77.1, I79.0, I79.2, K55.1, K55.8, K55.9, Z95.8, Z95.9 |
| Cerebrovascular disease |  | G45.x, G46.x, H34.0, I60.x–I69.x |
| Dementia |  | F00.x–F03.x, F05.1, G30.x, G31.1 |
| Chronic obstructive pulmonary disease |  | I27.9, J40.x–J47.x, J60.x–J67.x, J68.4, J70.1, J70.3 |
| Rheumatic disease |  | M05.x, M06.x, M31.5, M32.x–M34.x, M35.1, M35.3, M36.0 |
| Liver disease |  | B18.x, K70.0–K70.3, K70.9, K71.3–K71.5, K71.7, K73.x, K74.x, K76.0, K76.2–K76.4, K76.8, K76.9, Z94.4, K71.1, K72.1, K72.9, K76.5, K76.6, K76.7, I85.0, I85.9, I86.4, I98.2, K70.4, |
| Diabetes without chronic complication |  | E10.0, E10.1, E10.6, E10.8, E10.9, E11.0, E11.1, E11.6, E11.8, E11.9, E12.0, E12.1, E12.6, E12.8, E12.9, E13.0, E13.1, E13.6, E13.8, E13.9, E14.0, E14.1, E14.6, E14.8, E14.9 |
| Diabetes with chronic complication |  | E10.7, E11.2–E11.5, E11.7, E12.2–E12.5, E12.7, E13.2–E13.5, E13.7, E14.2–E14.5, E14.7 |
| Hemiplegia or paraplegia |  | G04.1, G11.4, G80.1, G80.2, G81.x, G82.x, G83.0–G83.4, G83.9 |
| Renal disease |  | I12.0, I13.1, N03.2–N03.7, N05.2–N05.7, N18.x, N19.x, N25.0, Z49.0–Z49.2, Z94.0, Z99.2 |
| AIDS/HIV |  | B20.x–B22.x, B24.x |

ICD-10: International Classification of Diseases, 10^th^ Revision

Diabetes with/without chronic complication is combined in the RCS Charlson Comorbidity Score

**Supplementary Table S3**: Summary statistics of comorbidity amongst patients diagnosed with Diffuse Large B-cell lymphoma (n=30,078) or Follicular lymphoma (n=15,551) in England during 2005-2013.

|  | **Diffuse Large B-cell Lymphoma** | | |  | **Follicular Lymphoma** | | |
| --- | --- | --- | --- | --- | --- | --- | --- |
|  | **None** | **Comorbidity** | **Multi-morbidity** |  | **None** | **Comorbidity** | **Multi-morbidity** |
|  |  |  |  |  |  |  |  |
| **Age**  **(y, SD)** | 66.9 (15.2) | 72.5 (12.6) | 73.0 (12.8) |  | 63.4 (13.6) | 70.2 (11.7) | 72.3 (11.0) |
|  |  |  |  |  |  |  |  |
| **Gender** |  |  |  |  |  |  |  |
| *Male* | 14,470 (53.9) | 815 (51.4) | 986 (60.7) |  | 6,792 (47.3) | 281 (43.8) | 261 (48.8) |
| *Female* | 12,398 (46.1) | 770 (48.6) | 639 (39.3) |  | 7,582 (52.8) | 361 (56.2) | 274 (51.2) |
|  |  |  |  |  |  |  |  |
| **Ethnicity*** |  |  |  |  |  |  |  |
| *White* | 19,418 (94.1) | 1,236 (96.2) | 1,204 (92.1) |  | 10,165 (94.9) | 514 (96.4) | 397 (92.8) |
| *Other* | 1,218 (5.9) | 49 (3.8) | 103 (7.9) |  | 550 (5.1) | 19 (3.6) | 31 (7.2) |
| *Missing* | 6,232 (23.2) | 300 (18.9) | 318 (19.6) |  | 3,659 (25.5) | 109 (17.0) | 107 (20.0) |
|  |  |  |  |  |  |  |  |
| **Deprivation** |  |  |  |  |  |  |  |
| *Least* | 5,808 (21.6) | 300 (18.9) | 262 (16.1) |  | 3,358 (23.4) | 113 (17.6) | 81 (15.1) |
| *2* | 6,035 (22.5) | 323 (20.4) | 344 (21.2) |  | 3,314 (23.1) | 127 (19.8) | 84 (15.7) |
| *3* | 5,616 (20.9) | 334 (21.1) | 333 (20.5) |  | 3,040 (21.2) | 139 (21.7) | 123 (23.0) |
| *4* | 5,223 (19.4) | 343 (21.6) | 344 (21.2) |  | 2,671 (18.6) | 129 (20.1) | 132 (24.7) |
| *Most* | 4,186 (15.6) | 285 (18.0) | 342 (21.1) |  | 1,991 (13.9) | 134 (20.9) | 115 (21.5) |
|  |  |  |  |  |  |  |  |
| Percentages may not sum to 100.0% due to rounding  * Percentages are calculated based on observed data | | | | | | | |
